# Anemia in people on second line antiretroviral treatment in Lilongwe, Malawi: a cross-sectional study

**DOI:** 10.1186/s12879-018-2952-9

**Published:** 2018-01-15

**Authors:** McNeil Ngongondo, Nora E. Rosenberg, Christopher C. Stanley, Robertino Lim, Dennis Ongubo, Richard Broadhurst, Colin Speight, Robert Flick, Petros Tembo, Mina C. Hosseinpour

**Affiliations:** 1 UNC Project, Tidziwe Centre, Private Bag, A-104 Lilongwe, Malawi; 2 University of North Carolina Schools of Medicine and Public Health, Chapel Hill, North Carolina USA; 3 Tufts University School of Medicine, Boston, USA; 4 Tulane University School of Public Health and Tropical Medicine, New Orleans, Louisiana, USA; 5 Lighthouse Trust, Lilongwe, Malawi

**Keywords:** Anemia, Second line, Art, HIV, Cross-sectional

## Abstract

**Background:**

Anemia is common among people living with HIV infection and is frequently associated with poor quality of life and poor prognosis. It has been well described in antiretroviral naïve individuals and those on non-nucleoside reverse transcriptase inhibitor-based first line antiretroviral therapy (ART) regimens. However there is limited information on anemia for ART experienced individuals on protease inhibitor-based second line ART regimens in resource limited settings. Our objective was to describe the prevalence and risk factors of anemia in this ART experienced population in Malawi.

**Methods:**

We conducted a cross-sectional study using routine facility data at two HIV clinics in Lilongwe, Malawi. The analysis included individuals receiving protease inhibitor-based second line ART. Clinical and laboratory data were collected at routine clinic visits. We used descriptive statistics, two-sample t-tests and multivariate logistic regression for data analysis.

**Results:**

Three hundred seventy-seven records were included in this analysis (37% male, median age 41 years, median CD4 count 415 cells/μL). The prevalence of anemia was 125/377 (33.2%) − mild, moderate and severe anemia was 17.5%, 13.8%, and 1.9% respectively. Female participants had a higher prevalence than male participants (43.6% vs. 15.7%, *p* < 0.001). In multivariate logistic regression, female sex (adjusted odds ratio (aOR) 5.3; 95% CI 2.9–9.5) and a CD4 count <200 cell/ul (aOR 3.1; 95%CI 1.6–6.0) were associated with increased risk of having anemia while a BMI ≥30 kg/m2 (aOR 0.8; 95% CI 0.6–1.0) and being on ART for more than 10 years (aOR 0.4; 95% CI 0.2–0.9) were associated with reduced risk of anemia. Being on a zidovudine- containing ART regimen was not associated with anemia.

**Conclusion:**

Anemia is common in people on second line ART in Lilongwe, Malawi. Screening for anemia in this population would be a useful strategy; especially for female patients, those who are underweight and have a low CD4 cell counts.

## Background

Anemia is common among individuals with Human Immunodeficiency Virus (HIV) infection [1]. Sub-Saharan Africa has a high prevalence of people living with HIV (PLHIV) with anemia. In these individuals anemia is associated with malnutrition, low CD cell counts and comorbidities such as tuberculosis [2, 3]. Whether the person is on ART or not, the presence of anemia predicts poor clinical outcomes such as death, clinical progression to AIDS, morbidity and a poor quality of life [4–6]. However, individuals who recover from anemia have better clinical outcomes [7].Therefore reducing anemia is a key component of care in people living with HIV.

Starting antiretroviral therapy (ART) improves hemoglobin levels and provides a protective effect against development of new anemia [8, 9]. As a result, people on ART have lower prevalence of anemia than those who are ART-naïve; underlining the value of ART in treating anemia [1, 5]. However in resource-limited settings data on the occurrence of anemia in PLHIV who have been on ART for several years. For PLHIV on second line ART regimens, the prevalence and risk factors of anemia are not well known even though persistent anemia continues to be clinically relevant while taking ART [10, 11].

As PLHIV are now living longer and HIV/AIDS programs across sub-Saharan Africa are maturing, more people are failing their first line ART regimens and are requiring second line ART regimens [11]. By 2016, approximately 1% of PLHIV (8811 PLHIV) in Malawi’s national ART program were on second line ART regimens and this number has been increasing every following year [12]. Therefore, studies need to address the gap in knowledge on anemia during long term ART treatment. HIV/AIDS programs should have strategies in place to improve survival and to reduce morbidity while people are on ART including strategies to identify those people at a greater risk of anemia during ART treatment.

We studied a cohort of ART-experienced Malawians on second line ART to describe the prevalence of and risk factors for anemia.

## Methods

### Study setting

We conducted a cross-sectional study using routine facility data from the Lighthouse (LH) and Martin Preuss Centre (MPC) HIV clinics in Lilongwe, Malawi, two tertiary referral ART clinics. The Lighthouse Trust, operating both LH clinic and MPC, is the largest provider of adult HIV care for PLHIV in Malawi. The integrated TB/HIV care program and Option B+ program for HIV positive pregnant and breastfeeding women and their families is based at MPC, while all other services are provided at both sites. ART regimens are standardized in the Malawi HIV/AIDS treatment guidelines [13]. Following first line ART regimen failure, patients start a second line ART regimen consisting of 2 NRTIs and a PI. The PIs used are atazanavir/ritonavir (ATV/r) and lopinavir/ritonavir (LPV/r). The NRTIs are tenofovir/lamivudine (TDF/3TC), zidovudine/lamivudine (AZT/3TC) and abacavir/lamivudine (ABC/3TC).

Stable individuals are seen at 3 month intervals for routine clinic visits. At these visits, a clinical assessment is done, tuberculosis is excluded by a 4 question symptom screen and cotrimoxazole prophylaxis and antiretroviral treatment are supplied. Viral load monitoring is done at 6 months following initiation of a new antiretroviral treatment regimen and then every 2 years thereafter per Malawi HIV/AIDS treatment guidelines [13]. There is no routine laboratory monitoring during ART.

### Study population

Study participants were identified from the clinics’ Electronic Medical Records (EMR) system. As part of implementation of the viral load monitoring policy at Lighthouse and Martin Preuss clinics, patients on second line ART for at least 6 months had viral load and safety laboratory tests. Adults (age ≥ 18 years) receiving PI-based second line antiretroviral regimens were included in this analysis.

### Study procedures

The data covers the period between October 2013 and April 2014. At their first routine clinic visits during this period, the study participants had clinical evaluations done and blood draws for full blood count (FBC), CD4 count, liver function tests, renal function tests, and viral load. Blood samples were processed at the UN-Project Laboratory in Lilongwe. VL was measured using the Abbott Real Ti*m*e HIV-1 assay system. CD4 cell counts were measured by flow cytometry using the Becton Dickinson FACSCount system (Becton Dickinson, Mountain View, California, USA). FBC was analyzed in the Beckman Coulter AcT 5diff Cap Pierce hematology analyzer (Beckman Coulter, Miami, FL), while liver and renal function tests were analyzed in the Roche Cobas C 311 chemistry analyzer.

The clinical evaluation and results of the laboratory tests were recorded into the EMR that is used at clinic visits. The EMR is used for recording clinical evaluations and for prescribing and dispensing drugs. Laboratory results, demographic information, antiretroviral treatment history, weight and height were extracted from the EMR into a database.

### Variables

The WHO hemoglobin concentrations for the diagnosis of anemia and assessment of severity were used to define anemia as hemoglobin (Hb) <12.0 g/dl for non-pregnant women and Hb <13.0 g/dl for men [14]. Anemia was further classified as mild (11–11.9 g/dl in non-pregnant women, 11–12.9 in men), moderate (8–10.9 g/dl for both sexes) and severe (<8 g/dl for both sexes) [14]. We used the WHO immunological classification for established HIV infection to classify HIV-associated immunodeficiency by CD4 counts: none or not significant (≥500 cells/μl), mild (350–499 cells/μl), advanced (200–349 cells/μl) and severe (<200 cells/μl) [15]. We used the WHO definition of virologic failure to define viral suppression as a viral load ≤1000 copies/ml [16]. Weight and height measurements were used to calculate the body mass index (BMI) which was classified as underweight (<18.5 kg/m^2^), normal (18.5–24.9 kg/m^2^), overweight (25–29.9 kg/m^2^) and obese (≥30 kg/m^2^). Serum creatinine, age, and sex were used to calculate the estimated glomerular filtration rate (GFR) using the 4-V MDRD eq. [17]. The mean corpuscular volumes (MCV) and mean corpuscular hemoglobin (MCH) were classified using the local UNC Project Laboratory reference values. The MCV and MCH were used to classify anemia.

### Statistical analysis

We used descriptive statistics to describe the population and to obtain the prevalence of anemia. We compared the means of the hemoglobin, age, total time on ART, time on second line ART, BMI and GFR between those with anemia and without anemia using two-sample t-tests. The categorical variables sex, second line regimen, viral load, CD4 count, MCV and RDW were analyzed using Chi-square and Fishers exact tests. Bivariable and multivariable logistic regression was used to estimate odds ratios (OR) for factors associated with anemia. All predictor variables were entered into a bidirectional stepwise variable selection multivariate model to identify the best set of risk factors for anemia. *P*-values for inclusion and removal of a variable were set at 0.05 and 0.1 respectively. We used a *p*-value of <0.05 to test significance and 95% confidence intervals (CI) to estimate precision. All statistical analyses were done using STATA SE version 12.1 (College Station, Texas).

## Results

Three hundred ninety-two individuals on second line ART were enrolled in the study. 5 records were excluded from the analysis because the individuals were not on second line ART; they had been erroneously included and their records showed they were not on second line ART during data analysis. An additional 10 records were excluded because the age was less than 18 as per inclusion criteria for this analysis. Therefore 377 records were included in the analyses.

Approximately two-thirds (237/377) of the study participants were female (Table 1). This is consistent with the ratio of men/women in the study clinic so women are not overrepresented. The average age of the participants was 41.9 years (SD = 9.6). More than three quarters of the participants (289/377) had been receiving ART for more than 5 years; the mean duration of ART use was 7.4 years (SD = 2.8). Anemic participants were significantly younger (40.5 years vs. 42.6 years, *p*-value = 0.01) and had been on second line ART for a shorter duration (2.9 years vs. 3.7 years, p-value = 0.004) than the non-anemic participants. There were no pregnant women.

**Table 1:** Characteristics of HIV-infected patients on second line ART in two urban HIV clinics in Lilongwe, Malawi, means ±SD or n (%)

Characteristic	Anemic	Not anemic	*P*-value†
	*n* = 125^a^	*n* = 252^a^	
Sex (%)			
Male	22 (17.6)	118 (46.8)	<0.001
Female	103 (82.4)	134 (53.2)	
Age, mean (SD) years	40.5 (9.4)	42.6 (9.6)	0.01
Total time on ART, mean (SD) years	7.1 (2.5)	7.5 (3.0)	0.17
Time on second line ART, mean (SD) years	2.9 (2.0)	3.7 (2.4)	0.004
Second line regimen (%)			
Contains AZT^b^	19 (15.2)	48 (19.1)	0.37
Does not contain AZT	106 (84.8)	204 (81.0)	
CD4 count (×10^3^cells/ul)			
> 500	42 (35.0)	89 (35.7)	0.003
350–499	30 (24.0)	72 (28.9)	
200–349	23 (18.4)	61 (24.5)	
< 200	30 (24.0)	27 (10.8)	
Viral suppression (copies/ml)			
< =1000	110 (89.4)	230 (91.6)	0.365
> 1000	13 (10.6)	21 (8.4)	
Body mass index, mean (SD) kg/m^2^	24.2 (5.1)	25.1 (5.2)	0.17
Estimated glomerular filtration rate	91.5 (31.0)	91.8 (29.9)	0.17

Anemia defined as Hb <12.0 g/dl for non-pregnant women and Hb <13.0 g/dl for men

^a^Missing values not included in the totals

^b^AZT- zidovudine

†*p*-value for t-test or chi-squared test as appropriate

**Fig. 1:**
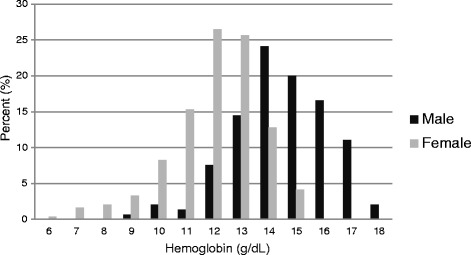
Distribution of hemoglobin among HIV infected patients on second line ART in male and female participants at 2 urban HIV clinics in Lilongwe, Malawi

The distribution of hemoglobin values between male and female participants is shown in Fig. 1. Men had higher hemoglobin values (median of 14.5 mg/dl; interquartile range (IQR) 13.6–15.5 mg/dl) than women (median of 12.3 mg/dl; IQR 11.1–13.1 mg/dl).

The prevalence of anemia was 125/377 (33.2%). Female participants had a higher prevalence than male participants (43.6% vs. 15.7%, *p* < 0.001). There were no differences in the total time on ART, time on second line ART and participants with suspected virologic failure on second line ART between male and female participants (data not shown).

The prevalence of mild, moderate and severe anemia was 66/377 (17.5%), 55/377 (13.8%) and 7/377 (1.9%) respectively. Among people who had anemia, most of the cases had normocytic (87/125) and normochromic (98/125) anemia (Table 2). A few cases had microcytosis (13/125) and macrocytosis (25/125). All 7 cases with severe anemia were female; 6 of these cases had microcytic, hypochromic anemia while 1 case had normocytic, normochromic anemia. 48/52 cases with moderate anemia were female participants while only 4 cases with moderate anemia were male participants. Most men with anemia (18/22 of men with anemia) had mild anemia.

**Table 2:** Description of the type and severity of anemia, *n* = 125

		Mild	Moderate	Severe	*P*-value†
		*(N = 66)*	*(N = 52)*	*(N = 7)*	
sex					0.002
	Male	18 (27.3)	4 (7.7)	0 (0.0)	
	Female	48 (72.7)	48 (92.3)	7 (100)	
MCV					<0.001
	<71	1 (1.5)	6 (11.5)	6 (85.7)	
	71–95	51 (77.3)	35 (67.3)	1 (14.3)	
	>95	14 (21.2)	11 (21.2)	0 (0.0)	
MCH					<0.001
	<23	0 (0.0)	9 (17.3)	6 (85.7)	
	23–34	62 (93.9)	35 (75.0)	1 (14.3)	
	>34	4 (6.1)	4 (7.7)	0 (0.0)	

MCV− mean corpuscular volume

MCH− mean corpuscular hemoglobin

†p-value for chi-squared test

During multivariate logistic regression female sex (adjusted odds ratio (aOR) = 4.1; 95% CI 2.5–7.0) and a CD4 count <200 cell/ul (aOR = 2.4; 95% CI 1.3–4.4) were associated with increased risk of having anemia while being on ART for more than 10 years (aOR = 0.4; 95% CI 0.2–0.8) and having a BMI ≥ 30 kg/m^2^ were associated with a reduced risk of anemia (Table 3). Age, viral suppression, glomerular filtration rate and the presence of AZT in the current regimen were not associated with anemia.

**Table 3:** Factors associated with anemia in HIV-infected patients on second line ART at two urban HIV clinics in Lilongwe, Malawi

	Unadjusted Odds Ratios		Adjusted Odds Ratios	
Characteristic	(95% CI)	*p*-value	(95% CI)	*p*-value
Sex				
Male	1		1	1
Female	4.1 (2.5–7.0)	<0.001	5.3 (2.9–9.5)	<0.001
Age				
≤29	1			
30–39	1.4 (0.6–3.2)	0.39		
40–49	0.9 (0.4–1.9)	0.69		
≥50	0.6 (0.3–1.5)	0.30		
Total time on ART (years)				
0–5	1		1	1
6–10	1.2 (0.7–2.0)	0.60		
11–15	0.4 (0.2–0.8)	0.02	0.4 (0.2–0.9)	0.02
Second line ART regimen				
contains AZT	1			
does not contain AZT	1.3 (0.7–2.4)	0.36		
CD4 cell count				
≥500	1		1	1
350–499	0.8 (0.5–1.6)	0.67		
200–349	0.8 (0.4–1.5)	0.47		
<200	2.4 (1.3–4.4)	0.01	3.1(1.6–6.0)	0.001
Viral load				
<=1000	1			
>1000	1.3 (0.6–2.7)	0.49		
Body mass index				
<18.5	1.5 (0.7–3.5)	0.33		
18.5–24.9	1		1	1
25–29.9	1.0 (0.6–1.8)	0.86		
≥30	0.6 (0.3–1.1)	0.08	0.8 (0.6–1.0)	0.02
Glomerular filtration rate				
>90	1			
30–89	0.7 (0.5–1.1)	0.15		
<30	9.0 (1–78.4)	0.047		

CI-confidence intervals

AZT- zidovudine

## Discussion

We found that anemia was common in this cohort on second line ART. The prevalence was highest in women who also had lower hemoglobin values and more severe degrees of anemia. Being female and a CD4 count <200 cell/ul were risk factors for anemia while BMI ≥30 kg/m^2^ and being on ART for more than 10 years were associated with reduced risk of anemia.

Although ART use is associated with a decrease in the prevalence of anemia, the prevalence in our study population was high [1, 8, 18]. Among the general adult population in Malawi, the prevalence of anemia ranges from 17% in men to 28% in non-pregnant women [19, 20]. Compared to this, we report a higher prevalence of 33.2% in people on second line ART− 15.7% in men and 43.6% in women. The prevalence of anemia in this study is similar to the prevalence observed in people on first line ART regimens where it was reported at 38.2% in a study of people on first line ART regimens [21]. However this prevalence is lower than the prevalence in ART naïve individuals which was reported at 77.4% in one study [22]. This is consistent with studies that show that ART reduces the prevalence of anemia in people living with HIV [21, 22].

The high prevalence of anemia in our study population is still worrying and points to a larger problem— that anemia remains untreated in large numbers of people on ART. This is a concern because anemia lowers the quality of life, an important treatment goal while on ART, and increases the risk of progression to AIDS and death [4–6]. The high prevalence of anemia highlights the need to address causes of anemia in people on ART.

The mechanism of anemia in this study population as in other populations living with HIV is multifactorial and includes both HIV-related and non-HIV related causes. The HIV-related causes are HIV itself which causes anemia of chronic disease due to chronic inflammation; opportunistic infections such as mycobacteria and fungi and neoplasms such as lymphoma which infiltrate bone marrow and inhibit maturation of progenitor cells as well as medications used during treatment of HIV and associated conditions such as zidovudine and cotrimoxazole [3, 7]. Treating HIV-related anemia should be with effective ART; appropriate treatment for the opportunistic conditions and removal of any suspected medications [1, 7, 23]. In our study, a few anemic participants (13/377) had VL >1000 cps/ml and were suspected to have treatment failure while the rest of the participants had viral suppression. We did not have data on current and past medical history and drug history (other than the ART history). However since this was a very ART experienced cohort, it is possible that participants had a significant history of opportunistic conditions and drugs that can cause anemia.

Endemic causes of anemia play a role in both the general population and in people living with HIV. In sub-Saharan Africa, these are chronic malnutrition; nutritional deficiencies such as iron deficiency, vitamin B12 deficiency and folate deficiency; infections and parasite infestations such as malaria, schistosomiasis and hookworms; pregnancy and hemoglobin disorders such as thalassemia, other hemoglobinopathies and other rare congenital hematologic disorders [24–26]. In settings such as ours where the anemia prevalence is already high from endemic causes, HIV infection worsens pre-existing anemia through its effects of chronic inflammation and immunosuppression [27].

Despite iron deficiency being a common cause of anemia within African populations, there was no preponderance of microcytosis nor hypochromia in our study population to suggest that iron deficiency is an important cause of anemia in the study participants []. Other studies have reported high rates of iron deficiency in people living with HIV and in those on ART [26]. A possible reason for the low prevalence of iron deficiency anemia in our study population is that this was an urban population which is associatied with lower prevalence of iron deficiency that rural populations [25]. In addition, the study participants were continuously in care at the HIV clinics with access to clinical assessments and hemoglobin level checks. The participants had many opportunities to be diagnosed with anemia and to have iron supplementation. The WHO recommends presumptive iron supplementation, anti-helminthic and anti-malarial therapy to treat anemia in resource limited settings where large workloads for health workers and inadequate laboratory capabilities make diagnosing specific causes of anemia difficult [28]. This approach is recommended in the Malawi Standard Treatment Guidelines and in other African countries [29]. However, a study in Mozambique showed that there is need for additional efforts to find and treat specific causes of anemia in PLHIV including in those on ART [30].

Nearly half of the female participants in this study had anemia, consistent with studies that show high prevalence of anemia in women living with HIV. Women living HIV are at a higher risk of severe anemia than men [4, 30]. The high prevalence in the women also reflects an overall higher prevalence of anemia in women in the general population [2]. In addition, six out of the seven women that had severe anemia in this study had microcytosis that indicates iron deficiency anemia − all were given iron supplementation. In contrast, almost all men had mild anemia except for 4 cases with moderate anemia. Whether additional factors such as differential ART failure in women, contribution of anemia in failure of first line ART regimen or use of NNRTI for prevention of mother to child transmission (PMTCT) may have contributed to the high prevalence of anemia in women this study is not known. Women could benefit from routine screening for anemia before starting ART and routine hemoglobin monitoring while they are on ART.

There are several reasons for the high proportion of women in our study population. In Malawi, more women are infected with HIV than men; women tend to present for health services more frequently than men [20] and with the Option B+ program that has been implemented since 2011, pregnant women are systematically targeted for HIV testing and those who have HIV start life-long ART regardless of the CD4 cell count [13]. As a result, more women are on ART than men. Our study population has similar gender demographics with the overall clinic population so that women are not overrepresented in the study population. From our findings, there was no difference in time spent on first line ART, time on second line ART and in virologic suppression between men and women.

We did not see an association between AZT in the current second line ART regimen and anemia in our analysis. This is in contrast with the widely recognized risk of anemia reported in association with AZT use [4, 31, 32]. We suspect that the lack of an association was because participants who would have been anemic at initiation of ART were not started on an AZT-based ART regimen and those that were suspected of having AZT associated anemia would have already been switched from AZT before these data were collected. Huffam et al. also reported that prior ART experience could be protective against subsequent development of AZT associated anemia in later regimens [33]. This is relevant to this study population that had significant prior ART experience. Participants in this study spent an average of 7.4 years on ART. In addition, some studies have reported that AZT is not a significant risk factor for anemia when it is used as part of a combinational ART regimen as is the case in our study population [21, 29].

In this study, we described anemia in among an ART-experienced cohort on PI-based second line ART. A strength of this analysis is that it was conducted on a well-characterized cohort that is generalizable because it used data collected during routine clinic visits.

An important limitation of the study is the lack of data on other known risk factors for anemia such as the presence of fever, opportunistic infections (e.g. TB, oral candidiasis) and concurrent use of other medication which were not measured and were not included in the analysis [27]. When analyzing the data on AZT, we were not able to look at substitutions that were made prior to the date of sample collection. This could have the effect of confounding by indication the significance of AZT in the ART regimens. Because Hb monitoring is not routinely done for patients on ART outside of the two facilities included in this study, the prevalence reported may be different than that in the wider HIV-infected population on second line ART.

## Conclusion

The study shows that anemia is present at a high prevalence in ART experienced people on second line ART regimens. The study further shows the need to identify and treat the causes of anemia in addition to giving them ART. As would be expected, female patients, those with CD4 cell count less than 200cells/ul were at higher risk of anemia while those who were overweight had lower risk of anemia. Further longitudinal follow-up studies are needed to explore long term hemoglobin changes after antiretroviral treatment is initiated and to find the specific causes of anemia in people on ART.

## Abbreviations

4-V MDRD: 4-Variable Modification of Diet in Renal Disease equation
ABC/3TC: Abacavir/lamivudine
AIDS: Acquired Immunodeficiency Syndrome
aOR: Adjusted Odds Ratio
ART: Antiretroviral Therapy
ATV/r: Atazanavir/ritonavir
AZT: Zidovudine
BMI: Body Mass Index
CI: Confidence Interval
EFV: Efavirenz
EMR: Electronic Medical Record
FBC: Full Blood Count
GRF: Glomerular filtration rate
Hb: Hemoglobin
HIV: Human Immunodeficiency Virus
HIV-RNA: Human Immunodeficiency Virus Ribonucleic Acid
IRB: Institutional Review Board
LH: Lighthouse HIV Clinic
LPV/r: Lopinavir/ritonavir
MCH: Mean corpuscular hemoglobin
MCV: Mean corpuscular volume
NHSRC: National Health Sciences Research Committee
NNRTI: Non-Nucleoside Reverse Transcriptase Inhibitor
NRTI: Nucleoside Reverse Transcriptase Inhibitor
NVP: Niverapine
OR: Odds Ratio
PI: Protease Inhibitor
PLHIV: People living with HIV
PMTCT: Prevention of Mother to Child Transmission
RDW: Red cell Distribution Width
SD: Standard Deviation
SSA: Sub-Saharan Africa
TB: Tuberculosis
TDF/3TC: Tenofovir/lamivudine
TDF/3TC/EFV: Tenofovir/lamivudine/efavirenz
WHO: World Health Organization
ZDV/3TC: Zidovudine/Lamivudine
